# Lipid Peroxidation-Mediated Inflammation Promotes Cell Apoptosis through Activation of NF-*κ*B Pathway in Rheumatoid Arthritis Synovial Cells

**DOI:** 10.1155/2015/460310

**Published:** 2015-02-05

**Authors:** Geng Yin, Ying Wang, Xiao-min Cen, Min Yang, Yan Liang, Qi-bing Xie

**Affiliations:** Department of Rheumatology and Immunology, West China Hospital of Sichuan University, Chengdu, Sichuan 610041, China

## Abstract

Rheumatoid arthritis (RA) is a systemic autoimmune disease characterized by chronic inflammation of multiple joints. The central pathogenesis of RA is the proliferation of synovial fibroblasts in response to inflammatory cytokines. However, some of the targeted therapies for inflammation reactions do not display significant clinical improvement after initiation of therapy. Thus, the relationship between inflammatory responses and RA therapy is still incompletely understood. In the present study, we proposed to determine whether enhanced inflammations may lead to cell apoptosis in rheumatoid arthritis synoviocytes. Our results indicated that products of lipid peroxidations, 4-HNE, may induce synovial intrinsic inflammations by activating NF-*κ*B pathways and it may lead to cell apoptosis. Pharmacological inhibition of NF-*κ*B activation may reduce the 4-HNE mediated inflammation responses and subsequent cell apoptosis. Our results may help to clarify the role of inflammations on RA development and imply that blocking NF-*κ*B activation may be partly beneficial for human RA therapy. These findings might provide a mechanism-based rationale for developing new strategy to RA clinical therapy.

## 1. Introduction

Rheumatoid arthritis (RA) is a systemic autoimmune disorder with progressive articular damage that may result in lifelong disability [1, 2]. It typically causes a symmetrical chronic arthritis that causes joint pain, swelling and in some cases a systemic illness [3, 4]. The central pathogenesis of rheumatoid arthritis (RA) is characterized by not only marked hyperplasia of the fibroblast-like synoviocytes (FLSs) in response to the production of autocrine, but also paracrine molecules produced by infiltrating mononuclear cells [3, 5]. The aberrant hyperplastic FLSs display properties of transformed cells and destroy periarticular bone and cartilage [6]. Actually, FLSs actively participate in the invasive processes of RA [7]. Since FLSs proliferate abnormally, they produce high levels of destructive enzymes and cytokines to decrease the susceptibility to spontaneous and induced apoptosis [8]. Thus, it is important to elucidate the mechanisms of FLSs proliferation and apoptosis for the design of targeted drugs.

Notably, because RA is a systemic autoimmune disease characterized by chronic inflammation of multiple joints and proinflammatory cytokines such as tumour necrosis factor (TNF)-*α*, interleukin (IL)-1*β*, IL-6, or IL-17 may be reconsidered as the etiology for RA and further leads to the development of targeted therapies [3, 9–11]. Indeed, TNF-*α* inhibitors, anti-TNF antibodies, a soluble TNF receptor fusion protein, and IL-1 receptor antagonist have been attempted for the treatment of RA, but their side effects such as serious infections and inducible malignant tumors still remain not to be resolved [10]. Moreover, about 25~30% of RA patients treated with TNF antagonists do not display any significant clinical improvement after initiation of therapy [11]. Patients will display feature of secondary resistance to the delivered drug within the first 5 years of therapy [10]. All these findings raise a question that it is still incompletely understood on the relationship between inflammation inhibitions and RA therapy.

In mammalian cells, nuclear factor-*κ*B (NF-*κ*B) is a master regulator of inflammations. It includes subunits as NF-*κ*B1 (p50/p105), NF-*κ*B2 (p52/p100), RelA (p65), c-Rel, and RelB and regulates immune and inflammatory responses, cellular proliferation, or cell death [12, 13]. NF-*κ*B is constitutively activated in many autoimmune diseases, including RA [14, 15]. For example, FLSs release cytokines, chemokines, and growth factors to sustain inflammation and produce enzymes that degrade the organized extracellular matrix, destroying cartilage and bone [16, 17]. Moreover, NF-*κ*B activation facilitates synovial hyperplasia by promoting proliferation and inhibiting apoptosis of FLSs [18]. Thus, NF-*κ*B seems to be a positive regulator of cell growth and inhibitor of cell apoptosis in FLSs. However, some studies have suggested that NF-*κ*B may also function in a proapoptotic fashion. For example, induction of apoptosis in human embryonic kidney cell line (293 cells) after serum withdrawal requires NF-*κ*B activation [19]. Similarly, inhibition of NF-*κ*B activation prevents apoptosis in cultured human thymocytes [20]. NF-*κ*B activation may promote apoptosis in neural cells [21] and T cell hybridomas [22], and high levels of c-Rel induce apoptosis in avian embryos and in bone marrow cells* in vitro* [23]. Thus, the functions of NF-*κ*B in apoptosis may be distinct by different cell types. At least, all the paradox indicates that inflammations mediated by NF-*κ*B may not always be beneficial for cell survival. Ectopic elevations of inflammations may afford us new insights into the clinical RA therapy and explain why sometimes anti-inflammation drugs are not so efficacious.

In this study, we proposed to determine whether enhanced inflammations may lead to cell apoptosis in FLSs. Thus, we screened and identified the inflammation alternations by 4-hydroxynonenal (4-HNE) treatment in MH7A cells, a human rheumatoid arthritis synovial cell line. 4-HNE is an aldehyde product of membrane lipid peroxidation, generated through peroxidation of omega 6-polyunsaturated fatty acids [24, 25]. Our results showed that, by 4-HNE treatment, FLSs develop strong inflammation reactions, such as elevated IL1-*β*, IL-6, and TNF-*α* transcriptions and COX-2 expressions. Of note, 4-HNE induces NF-*κ*B activation, indicated by RelA (p65) nuclear translocations and subsequent cell apoptosis. However, inactivation of NF-*κ*B leads to reduced inflammations and cell apoptosis. The present findings may help to understand the molecular network of inflammations and apoptosis in FLSs and offer novel insights into the clinical improving of RA therapy.

## 2. Material and Methods

### 2.1. Reagents

The 4-hydroxynonenal was obtained from Biomol (Plymouth Meeting, PA, USA). Dulbecco's Modified Essential Medium (DMEM), Fetal Bovine Serum (FBS), and BAY11-7082 (NF-*κ*B inhibitor) were purchased from GIBCO Invitrogen (Carlsbad, CA, USA). The Millipore Luminex kits for cell signaling multiplex assay were from Millipore (Billerica, MA, USA) (#48-611MAG and 48-680MAG). The Hoechst kit for cell death detection was from Beyotime Biotechnology (Haimen, Jiangsu, China). The following antibodies, anti-COX-2, anti-Lamin A/C, anti-beta actin, and anticleaved caspase 3 were from Santa Cruz Biotechnology (Santa Cruz, CA, USA). The NF-*κ*B (p65) and GAPDH antibodies were from Cell Signaling Technology (Danvers, MA, USA). Other chemicals were of the highest purity available.

### 2.2. Cell Culture

A widely used MH7A human rheumatoid arthritis synovial cell was chosen as* in vitro* experiment system [2], which was obtained from Shanghai Institute of Cell Biology (Introduced from American Type Culture Collection). In our experiments, MH7A cells were plated in 6-well plates at 1.0 × 10^6^ cells/mL. The cells were incubated in Dulbecco's Modified Essential Medium (DMEM) containing 10% Fetal Bovine Serum (FBS) plus antibiotics for 24 h in 5% CO_2_ at 37°C. For 4-HNE and following BAY11-7082 treatment, the final low and high concentrations (5 *μ*M and 50 *μ*M) of 4-HNE and 10 *μ*M BAY11-7082 were applied to these cells and then incubated from 0 to 12 h. No additives were used as internal controls. After culturing, the cells were harvested for subsequent examinations.

### 2.3. Cell Signaling Multiplex Assay

To screen the targeted intracellular signaling of 4-HNE on MH7A cells, cell signaling multiplex assay was carried out by Millipore Luminex kits. Briefly, MH7A cells were treated by 4-HNE and then lysed in lysis buffer. The cell samples were loaded to 96 wells and coated with respective primary antibody mixtures. After washing, samples were detected in Luminex Multiplexing Instruments (Millipore, Billerica, MA).

### 2.4. Quantitative Real-Time PCR

Total RNA was extracted from tissues using TRizol reagent (Invitrogen). RNA was subjected to reverse transcription with reverse transcriptase as the manufacturer's instructions (Fermentas). Quantitative real-time PCR was performed using the Bio-Rad iQ5 system, and the relative gene expression was normalized to internal control as Beta actin. Primer sequences for SYBR Green probes of target genes are as follows,* IL-1β *: CTGGTGTGTGACGTTCCCATTA and CCGACAGCACGAGGCTTT;* IL-6*: TTCCATCCAGTTGCCTTCTTG and TTGGG AGTGGTATCCTCTGTGA;* Tnf-α* : CATCTTCTCAAAATTCGAGTGACA and TGG GAGTAGACAAGGTACAACCC.

### 2.5. Western Blot

Western blot was performed as standard procedures. MH7A cell samples were lysed in lysis buffer, and insoluble material was removed by centrifugation at 12,000 g for 20 min at 4°C. Final protein concentrations were determined using the Bradford protein assay (Bio-Rad Laboratories). Electrophoresis was performed using SDS-PAGE, and blots were transferred to nitrocellulose membranes. Membranes were incubated with appropriate primary antibodies and secondary antibodies. Membranes were then visualized using an enhanced chemiluminescent technique. Resulting films were scanned, and optical densities were quantified using ImageJ.

### 2.6. NF-*κ*B Translocation and Activity Assays

To detect the NF-*κ*B nuclear translocations, MH7A cells were harvested and isolated into cytosol and nuclear parts, according to instructions of Nuclear/Cytosol Fractionation Kit (Biovision). As for the NF-*κ*B activity detections [13], plasmids encoding NF-*κ*B-responsive firefly luciferase and Renilla luciferase, which is an internal control for normalizing transfection, were transfected to MH7A cells using lipofectamine 2000. After transfection for 0, 3, or 12 h, cells were washed with phosphate-buffered saline, serum-starved in Opti-MEM overnight, and then assayed for luciferase activity using the dual-luciferase reporter assay kit (Promega).

### 2.7. Assay of Cell Death Detection

For the preparation of cell death detection by Hoechst staining, MH7A cells were plated with 1.0 × 10^5^ cells/mL in 6-well plates. After pharmacological manipulations, cells were directly stained with Hoechst kit from Beyotime. The cell counting was carried out through the use of National Institutes of Health software ImageJ, which is available from http://rsbweb.nih.gov/.

### 2.8. Statistical Analysis

Data represent the mean and standard error of the mean (SEM). Student's *t*-test was performed for all statistical significance analysis using GraphPad Prism software. ^∗^ *P* < 0.05, ^∗∗^ *P* < 0.01, and ^∗∗∗^ *P* < 0.001.

## 3. Results

### 3.1. Effects of 4-HNE on Multiple Intracellular Pathways in Rheumatoid Arthritis Synoviocytes

**Figure 1:**
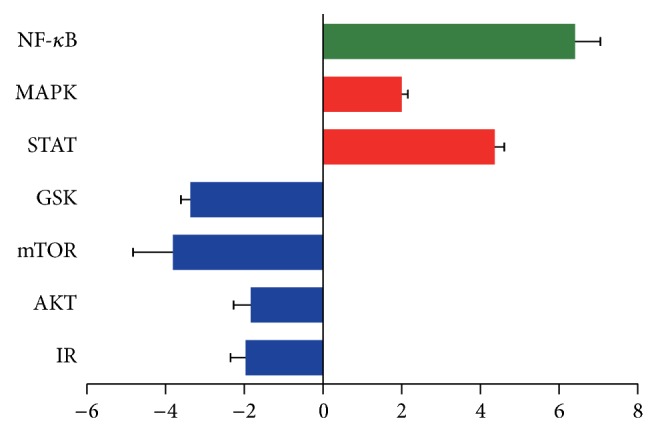
Alternations of multiple intracellular pathways by 4-HNE treatment in MH7A synovial cells. Histograms showing that multiple intracellular pathways are altered by 4-HNE treatment (5 *μ*M for 3 h) through Millipore Luminex assays. Noted that insulin receptor (IR), AKT, mTOR, and GSK pathways are downregulated by 4-HNE treatment (blow) and NF-*κ*B, MAPK, and STATs pathways are upregulated (red, NF-*κ*B green).

To screen the effects of lipid peroxidation on synoviocytes, we examined the intracellular pathways by Millipore Luminex kits after 4-HNE treatment. The alternations of multiple intracellular pathways in rheumatoid arthritis synovial cells were demonstrated below. It is noted that, after 4-HNE treatment, inflammation relative pathways, such as NF-*κ*B and STATs, were significantly upregulated (Figure 1), while the cell growth or survival relative pathways, such as insulin receptor (IR), AKT, mTOR, and GSK pathways, were downregulated (Figure 1). These results are consistent with the previous findings, indicating that lipid peroxidation is able to induce cell stress to promote inflammations and inhibit cell growth. As for the ectopic upregulation of inflammations by 4-HNE treatment, we determine to identify the subsequent inflammation alternations and cell viability in MH7A synoviocytes.

### 3.2. Lipid Peroxidations Activate Inflammation Reactions in Rheumatoid Arthritis Synoviocytes

**Figure 2:**
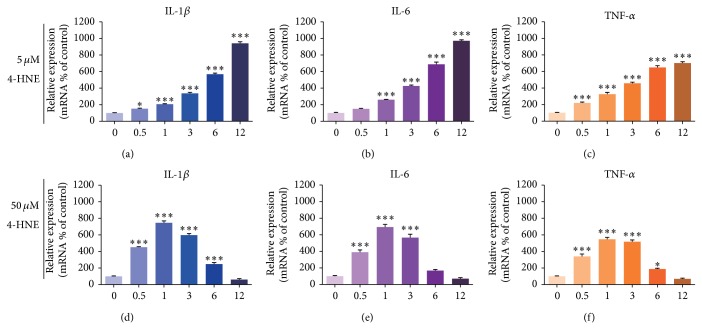
Activated inflammation reactions by 4-HNE treatment in MH7A synovial cells. ((a)–(c)) Real-time PCR results showing the increased mRNA levels of inflammation factors:* IL1-β* (a),* IL-6* (b), and* Tnf-α* (c) in MH7A rheumatoid arthritis synovial cells after 4-HNE treatment of low concentration (5 *μ*M) for 0~12 h. ((d)–(f)) Real-time PCR results showing the mRNA levels of inflammation factors:* IL1-β* (d),* IL-6* (e), and* Tnf-α* (f) are increased (0~3 h) and then decreased (3~12 h) in MH7A rheumatoid arthritis synovial cells after 4-HNE treatment of high concentration (50 *μ*M). Results are averages of five independent experiments. Data represent mean ± SEM. ^∗^ *P* < 0.05, ^∗∗^ *P* < 0.01, and ^∗∗∗^ *P* < 0.001.

To further confirm the inflammation inductions by 4-HNE treatment, we next examined the expressions of canonical inflammation factors, including IL1-*β*, IL-6, and TNF-*α* [3]. The results of quantitative real-time PCR showed that under low concentrations of 4-HNE (5 *μ*M), the mRNA levels of IL1-*β*, IL-6, and TNF-*α* were increased gradually with the increasing time up to 12 h, and the maximum folds increased by 6.79, 6.19, and 4.28 compared to the control, respectively (Figures 2(a), 2(b), and 2(c)). This confirms that lipid peroxidations may induce inflammations in rheumatoid arthritis synoviocytes. Interestingly, when the concentration of 4-HNE increased to 50 *μ*M, the mRNA levels of IL1-*β*, IL-6, and TNF-*α* in rheumatoid arthritis synovial cells increased gradually with the increasing time up to 1 h and then decreased (Figures 2(d), 2(e), and 2(f)). The shorter peak time of mRNA increasing may indicate that inflammations are enhanced by high lipid peroxidation treatment. And the overwhelmed lipid peroxidation may terribly affect normal cell functions and lead to a subsequent reduction of inflammation gene transcriptions. Nevertheless, our results support the notion that lipid peroxidation may indeed induce inflammation reactions in synoviocytes.

### 3.3. Lipid Peroxidations Induce COX-2 Expression in Rheumatoid Arthritis Synoviocytes

**Figure 3:**
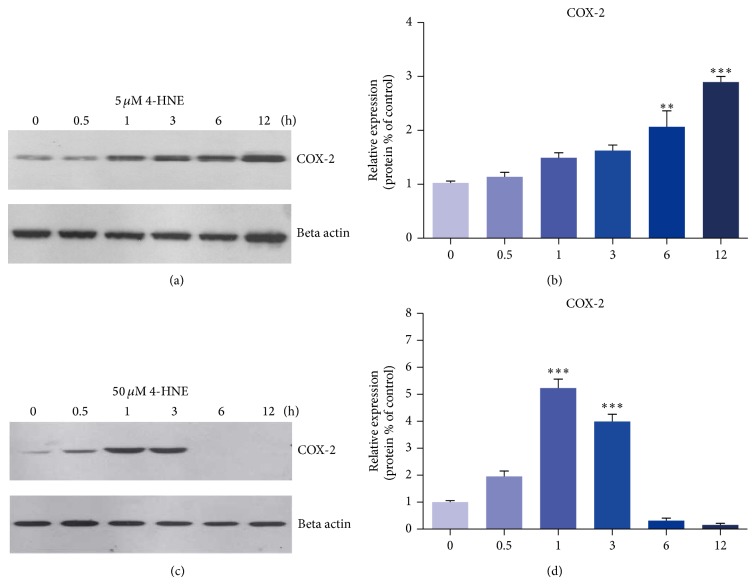
Induced COX-2 expression by 4-HNE treatment in MH7A synovial cells. ((a)-(b)) Western blots and histograms showing that the protein levels of COX-2 are induced by 4-HNE treatment of low concentration (5 *μ*M) for 0~12 h. ((c)-(d)) Western blots and histograms showing that the protein levels of COX-2 are increased by 4-HNE treatment of high concentration (50 *μ*M) for 0~3 h and dramatically decreased in the following 3~12 h. Results are the averages of three independent experiments. Data represent mean ± SEM. ^∗^ *P* < 0.05, ^∗∗^ *P* < 0.01, and ^∗∗∗^ *P* < 0.001.

To further confirm the inflammation alternations by lipid peroxidation, we next examined the protein level of COX-2 in 4-HNE-treated MH7A cells. COX-2 is an inducible isoform of prostaglandin H synthase, which mediates prostaglandin synthesis during inflammations [26]. We found that, under 5 *μ*M 4-HNE conditions, COX-2 protein levels were increased gradually with the time up to 12 h, with the highest value being about 2.86-folds that of the control (Figures 3(a) and 3(b)). This indicates that lipid peroxidations may induce COX-2 expressions in a time-dependent manner in MH7A synovial cells. When the 4-HNE concentration came to 50 *μ*M, the protein levels of COX-2 were increased gradually with the increasing time up to 1 h and then decreased (Figures 3(c) and 3(d)). These alternations are consistent with the changes of inflammation gene transcriptions, suggesting that lipid peroxidations could enhance inflammation reactions in rheumatoid arthritis synoviocytes.

### 3.4. Lipid Peroxidations Activate NF-*κ*B Pathway in Rheumatoid Arthritis Synoviocytes

**Figure 4:**
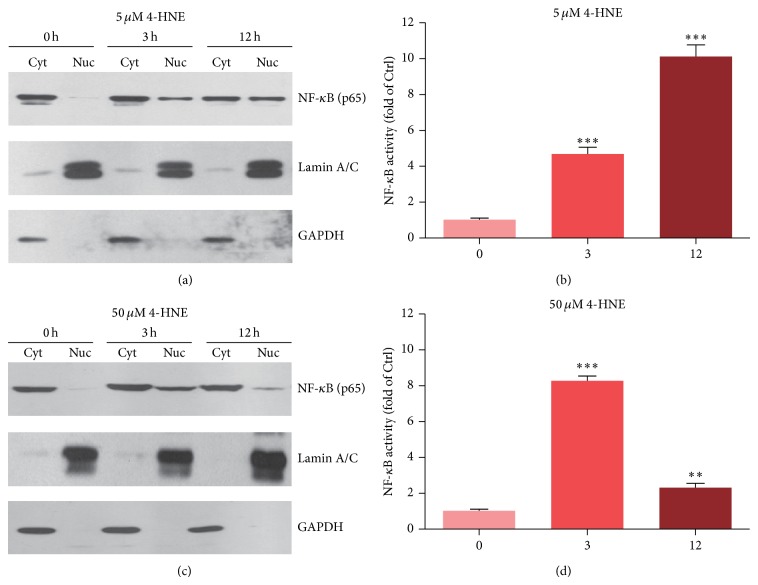
Activated NF-*κ*B pathway by 4-HNE treatment in MH7A synovial cells. ((a)-(b)) Western blots and histograms showing that the nuclear translocations of NF-*κ*B (p65) are increased by 4-HNE treatment of low concentration (5 *μ*M) for 3 and 12 h. Cyt: cytosol; Nuc: nuclear. ((c)-(d)) Western blots and histograms showing that nuclear translocation and activity of NF-*κ*B (p65) are increased by 4-HNE treatment of high concentration (50 *μ*M) for 3 h and decreased in 12 h. Results are averages of three independent experiments. Data represent mean ± SEM. ^∗^ *P* < 0.05, ^∗∗^ *P* < 0.01, and ^∗∗∗^ *P* < 0.001.

Inflammation is controlled by multiple signaling pathways in mammalian cells, and NF-*κ*B plays a pivotal role in the regulation of inflammatory responses. By Millipore Luminex kits, we have identified that NF-*κ*B pathway is induced by 4-HNE. To confirm this finding, we further investigated the NF-*κ*B activations by 4-HNE treatment. Upon activation of NF-*κ*B by stimulators, NF-*κ*B subunits (especially for p65) translocate into the nucleus to activate transcription of genes involved in cell inflammations or proliferations [27]. Accordingly, we evaluated NF-*κ*B activity by detecting the nuclear localization of the p65 subunit. Subcellular fractionation of low 4-HNE-treated MH7A cells showed that p65 localization was enhanced in the nucleus compared to controls (Figure 4(a)). An NF-*κ*B-RE-Luc reporter assay also demonstrated that NF-*κ*B activity was 10.1-fold greater after 12 h 4-HNE treatment compared with the level in controls (Figure 4(b)). Under high 4-HNE concentrations, NF-*κ*B translocation into nucleus was increased as early as 3 h but decreased at 12 h (Figure 4(c)). The NF-*κ*B activity assay also exhibited that NF-*κ*B activity was increased in 3 h and decreased in 12 h, respectively. These changes are highly correlated with the quantitative changes of inflammation assayed above. Thus, these results indicate that NF-*κ*B activity could be induced by lipid peroxidations, and overwhelmed oxidative stress may even impair the NF-*κ*B activation conversely.

### 3.5. Lipid Peroxidations Induce Apoptosis in Rheumatoid Arthritis Synoviocytes

**Figure 5:**
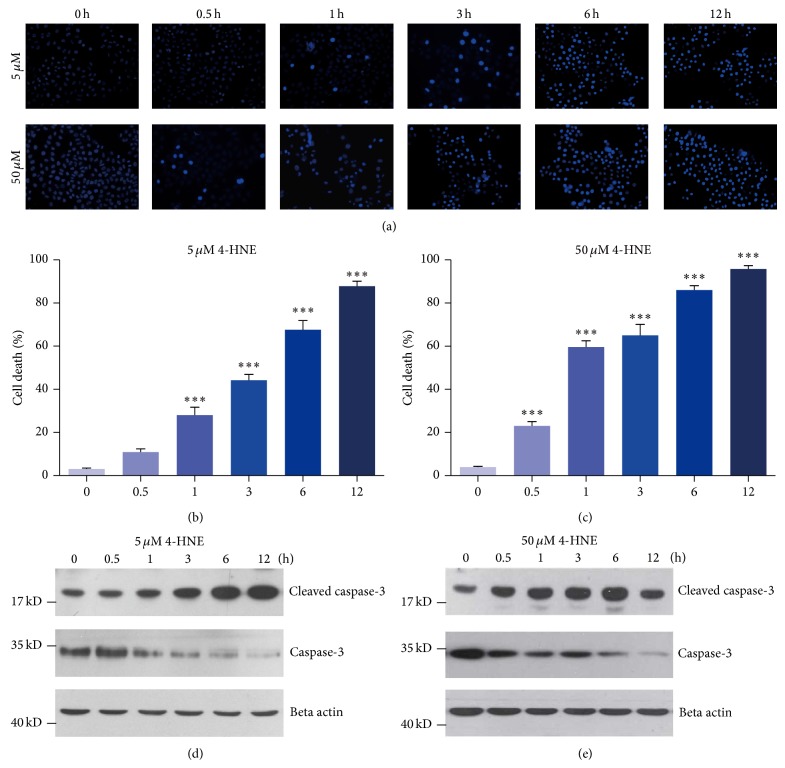
Dramatic apoptosis by 4-HNE treatment in MH7A synovial cells. ((a)–(c)) Representative images and histograms showing that percentages of cell apoptosis are increased by 4-HNE treatment of both low concentration (5 *μ*M) ((a) and (b)) and high concentration (50 *μ*M) ((a) and (c)) groups from 0 to 12 h. Results are averages of five independent experiments. Data represent mean ± SEM. ^∗∗∗^ *P* < 0.001. ((d)-(e)) Western blots showing that protein levels for apoptotic marker, cleaved caspase 3, are increased by 4-HNE treatment of both low concentration (5 *μ*M) (d) and high concentration (50 *μ*M) (e) groups from 0 to 12 h. Note that in the high concentration group, cleaved caspase 3 begins to decrease in 12 h compared to that of 6 h.

Lipid peroxidation has been proved to induce NF-*κ*B pathway and inflammatory responses in MH7A cells; we wonder how these inflammations affect cellular functions, especially on cell viability. To examine whether 4-HNE treatment may induce the cell apoptosis in MH7A cells, we applied Hoechst staining to MH7A cells treated by 4-HNE. Quantitative results showed that 5 *μ*M 4-HNE could induce dramatic cell apoptosis in MH7A cells, with nearly 87.8% cell apoptosis in the time of 12 hours (Figures 5(a) and 5(b)). Comparably, high concentration 4-HNE induces faster cell apoptosis than that in low 4-HNE group (Figures 5(a) and 5(c)). We also examined cleaved caspase 3, a well-known apoptotic marker to confirm the cell apoptosis by 4-HNE. Results showed that the cleaved caspase 3 increased as 4-HNE treatment in a time- and dose-dependent manner. Moreover, our findings also indicated that the cleaved caspase 3 increased and total caspase 3 decreased as 4-HNE treatment in a time- and dose-dependent manner (Figures 5(d) and 5(e)). It is noted that the protein level of cleaved caspase 3 seemed to decrease in 50 *μ*M 4-HNE for 12 h, which could be explained as the solemn impairment of cell structures and functions for overwhelming 4-HNE treatment. Based on the above results, these findings suggested that lipid peroxidation may induce apoptosis in rheumatoid arthritis synoviocytes.

### 3.6. Inhibition of NF-*κ*B Activation May Reduce Inflammations in Rheumatoid Arthritis Synoviocytes

**Figure 6:**
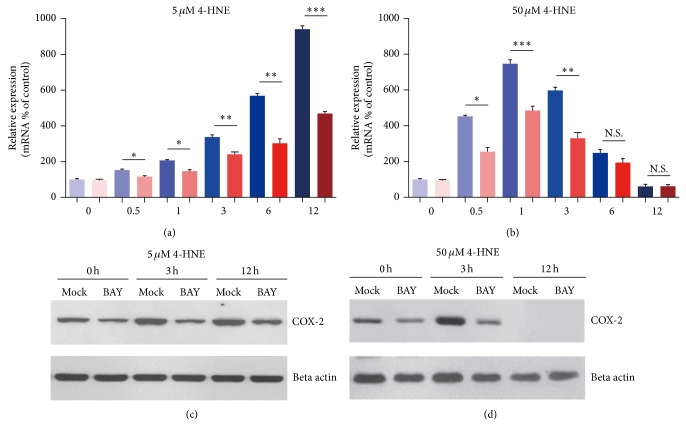
Inhibition of NF-*κ*B activation reduces inflammation in MH7A synovial cells. ((a)-(b)) Histograms showing mRNA levels of* IL1-β* are reduced by BAY11-7082 (NF-*κ*B inhibitor) treatment (10 *μ*M) under the condition of 4-HNE treatment of low concentration (5 *μ*M, 0~12 h) (a) and high concentration (50 *μ*M, 0~12 h) (b). Results are averages of five independent experiments. Data represent mean ± SEM. ^∗^ *P* < 0.05, ^∗∗^ *P* < 0.01, and ^∗∗∗^ *P* < 0.001. ((c)-(d)) Western blots showing protein levels of COX-2 are partly reduced by BAY11-7082 treatment (10 uM) under the condition of 4-HNE treatment of low concentration (5 *μ*M, 0~12 h) (c), while not altered in groups of high concentration (50 *μ*M, 0~12 h) (d).

Since NF-*κ*B is a master regulator of inflammatory responses, we would like to test whether inhibition of NF-*κ*B activation could reduce inflammations and subsequent cell apoptosis in MH7A cells. Firstly, we examined the inflammatory responses by BAY11-7082 (NF-*κ*B inhibitor) treatment in MH7A cells under the condition of 4-HNE. The mRNA levels of* IL1-β*, a marker of inflammatory response, was lower in the presence of BAY11-7082 compared to that of 4-HNE alone group, either in low or high concentration of 4-HNE group (Figures 6(a) and 6(b)). Moreover, the induction of COX-2 was also reduced by BAY11-7082 treatment (Figures 6(c) and 6(d)). These findings indicate that the inhibition of NF-*κ*B signaling attenuates inflammatory responses in rheumatoid arthritis synoviocytes.

### 3.7. Inhibition of NF-*κ*B May Partly Rescue Cell Death by HNE in Rheumatoid Arthritis Synoviocytes

**Figure 7:**
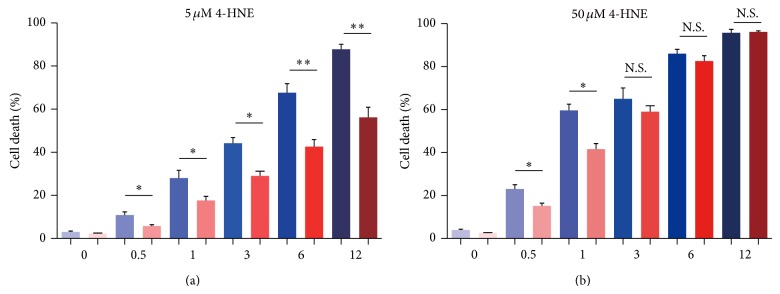
Inhibition of NF-*κ*B may partly rescue cell death by 4-HNE in MH7A synovial cells. Histograms showing that percentage of cell apoptosis are partly reduced by BAY11-7082 treatment (10 *μ*M) under the condition of 4-HNE treatment of low concentration (5 *μ*M, 0~12 h) (a) and high concentration (50 *μ*M, 0~3 h) (b). Results are averages of five independent experiments. Data represent mean ± SEM. ^∗^ *P* < 0.05, ^∗∗^ *P* < 0.01, and ^∗∗∗^ *P* < 0.001.

Since inhibition of NF-*κ*B activation could reduce inflammations in MH7A cells, we further investigate whether the NF-*κ*B inhibitor could reduce 4-HNE mediated apoptosis in MH7A cells. Quantitative results showed that in the presence of BAY11-7082, the percentage of cell apoptosis reduced compared to the counterparts without BAY11-7082 under 5 *μ*M 4-HNE conditions (Figure 7(a)). While in 50 *μ*M 4-HNE groups, the protective effect of BAY11-7082 was weakened, especially in 6 and 12 h groups (Figure 7(b)). This may be due to the solemn impairment of cell structures and functions by overwhelming lipid peroxidations. Nevertheless, these data at least verify that inhibition of NF-*κ*B and inflammations could partly reduce the 4-HNE mediated cell apoptosis in rheumatoid arthritis synoviocytes.

## 4. Discussion

**Figure 8:**
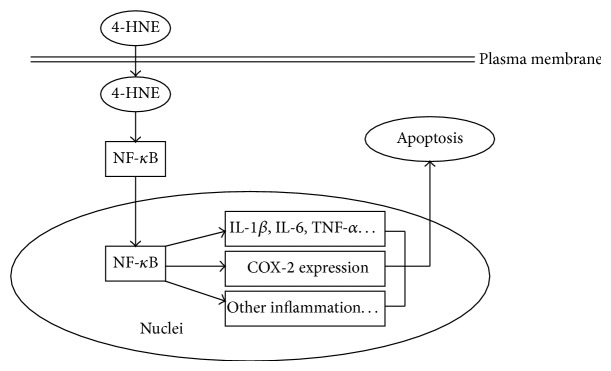
Schematic model showing the mechanism of lipid peroxidation toxicity in rheumatoid arthritis synovial cells. 4-HNE activates NF-*κ*B pathway by increasing nuclear translocation of NF-*κ*B. Then, intracellular inflammation pathways are activated, such as IL1-*β*, IL-6, TNF-*α*, and COX-2, to open the apoptotic cascade.

In the present study, we reveal a novel mechanism to clarify the role of inflammations on RA development. We demonstrate that products of lipid peroxidations, 4-HNE, could induce synovial intrinsic inflammations by activating NF-*κ*B pathways and lead to cell apoptosis. Pharmacological inhibition of NF-*κ*B activation may reduce the 4-HNE mediated inflammations and subsequent cell apoptosis (Figure 8). Our work uncovers novel relationships between inflammations and RA development and offers new strategy to RA clinical therapy.

Rheumatoid arthritis (RA) is a chronic inflammatory disease of synovium that can lead to severe joint damage [2]. The central pathogenesis of RA is a proliferation of fibroblast-like synoviocytes (FLSs) in response to stimulators. During the process of FLSs proliferation, inflammatory responses are critical for RA development [3]. Previous studies focus on drugs to inhibit inflammations, but they sometimes do not display significant clinical improvement after initiation of therapy, as well as bringing side effects such as serious infections and inducible malignant tumors [3]. The reason for this inefficacious phenomenon is not well understood. Now, our results could partly answer the question that why sometimes anti-inflammation drugs do not perform well in RA therapy. At least, the inhibitory effect of anti-inflammation drugs may partly block the inflammation-mediated cell apoptosis in rheumatoid arthritis synoviocytes. Therefore, appropriate drug compatibility should be important to balance the effect of inflammations on RA development and FLSs apoptosis.

Since inflammation balance is important for the survival of FLSs, the precise regulation of synovial inflammations must be well achieved. One of the central regulators is NF-*κ*B. It has been long appreciated that NF-*κ*B is a significant transcription factor that functions in immune and inflammatory responses [13]. For example, NF-*κ*B plays a pivotal role in myocardial ischemia-reperfusion injury and induces many proinflammatory cytokines and chemokines [14]. Moreover, NF-*κ*B is also thought to act as a redox sensitive transcription factor, that has been proposed to be the sensor for oxidative stress [28]. Reactive oxygen species (ROS) can activate NF-*κ*B directly [29], and ROS is also involved in NF-*κ*B activation by other stimuli such as TNF*α* [30]. In the present study, we demonstrate that products of lipid peroxidation, 4-HNE, may induce cell death in rheumatoid arthritis synovial cells by activating NF-*κ*B pathways. Our results confirmed that ROS and its relative products, that is 4-HNE, are enough to induce inflammation reactions* via* NF-*κ*B. Considering that ectopic activation of NF-*κ*B induces synovial apoptosis, it may afford us some new strategy to design antirheumatic drugs targeted at NF-*κ*B.

Multiple extracellular and intracellular stimulators may induce inflammation reactions, which may exacerbate RA. Most importantly, oxidative free radical or ROS is attributable to RA development. Besides the ROS itself, the products of lipid peroxidation may also cause the formation of reactive aldehydes and lead to inflammations. These lipid peroxidations have longer biological half-lives than free radicals and can diffuse from their site of formation to reach distant targets and cause cellular damage [31]. 4-HNE is an *α*, *β*-unsaturated hydroxyalkenal that is produced by lipid peroxidation in cells [32, 33]. It is found throughout animal tissue and in higher quantities during oxidative stress for the increase in the lipid peroxidation chain reaction and for the increase in stress events [34, 35]. However, the “normal” level of lipid peroxidation is limited to a low level, and even the modest upregulation could alter multiple intracellular pathways. Thus, we propose that lower levels of intracellular 4-HNE are beneficial to cells, but higher levels can cause a toxic response in the cell and may lead to cell death. In this study, we applied different doses of 4-HNE to treat MH7A synovial cells for different time points. Interestingly, we note that even a short overwhelmed treatment of 4-HNE exhibits pretty effects on synovial apoptosis. Notably, the effect of low concentration 4-HNE could be partly restored by NF-*κ*B inhibitors, while high concentration 4-HNE could not. This suggests that at least the effects of lipid peroxidation to synovial apoptosis might be partly dependent on NF-*κ*B mediated inflammation reactions.

## 5. Conclusion

In conclusion, the results of this study support the notion that lipid peroxidation-mediated inflammation promotes cell apoptosis through activation of NF-*κ*B pathway in rheumatoid arthritis synovial cells. 4-HNE treatment may induce synovial apoptosis by activating NF-*κ*B mediated inflammations. Inhibition of NF-*κ*B nuclear translocation may partly rescue the inflammation reactions and cell apoptosis. Our results imply that blocking NF-*κ*B activation may be partly beneficial for human RA therapy.
